# Intranasal Vaccination with a Recombinant Adeno-Associated Virus Type 6 Encoding SapM Confers Protection Against Tuberculosis

**DOI:** 10.3390/vaccines14030224

**Published:** 2026-02-28

**Authors:** Chaonan Xing, Wenfei Wang, Jiahuan Yang, Siwan Feng, Jiayi Xiao, Ningjian Cai, Siwei Mo, Yi Cai, Xinchun Chen, Chenyan Shi

**Affiliations:** 1 College of Biological Sciences, China Agricultural University, Beijing 100193, China; xingchaonan06@163.com; 2 Guangdong Provincial Key Laboratory of Infection Immunity and Inflammation, Department of Pathogen Biology, Shenzhen University Medical School, Shenzhen 518060, China; 13630450124@163.com (J.Y.); 2200243012@email.szu.edu.cn (S.F.); 2400243067@mails.szu.edu.cn (J.X.); cainingjian1121@163.com (N.C.); mosiwei@zmu.edu.cn (S.M.); caiyi0113@szu.edu.cn (Y.C.); 3 National Clinical Research Center for Infectious Diseases, Shenzhen Third People’s Hospital, The Second Affiliated Hospital, School of Medicine, Southern University of Science and Technology, Shenzhen 518112, China; shy_wenfei@hotmail.com

**Keywords:** tuberculosis, SapM, vaccine, AAV, mucosal

## Abstract

**Background**: Effective tuberculosis vaccines capable of inducing durable pulmonary immunity remain an unmet need. Mucosal vaccination strategies and rational antigen selection are increasingly recognized as critical for improving protection against aerosol *Mycobacterium tuberculosis* (*Mtb*) infection. **Objective**: The objective of this study was to establish an intranasal recombinant adeno-associated virus (rAAV) platform and evaluate SapM (Rv3310) as a mucosal TB vaccine antigen in mice. **Methods**: We established and optimized an rAAV production and purification platform suitable for intranasal immunization and applied it to deliver *Mtb* antigen SapM. Immunogenicity was assessed by lung mucosal T-cell responses (CD69/CD103) and IFN-γ production in the lungs and spleen after mycobacterial antigen stimulation. Protective efficacy was evaluated after aerosol H37Rv challenge by quantifying pulmonary bacterial burden and lung pathology compared with vector controls and BCG. **Results**: rAAV6-SapM was successfully produced and efficiently transduced antigen-presenting cells without inducing phenotypic maturation. Intranasal immunization in mice induced mucosal T-cell responses in the lungs and increased expression of tissue residency-related markers (CD69 and CD103). It also elicited a Th1-biased cellular immune response characterized by enhanced IFN-γ production in both the lungs and spleen in response to mycobacterial antigen stimulation. Upon aerosol challenge with virulent *Mtb* H37Rv, rAAV6-SapM-immunized mice exhibited a significant reduction in pulmonary bacterial burden and attenuated lung pathology compared with vector-immunized controls. **Conclusions**: These findings provide proof-of-concept evidence that intranasal delivery of an AAV-based vaccine encoding SapM can induce antigen-responsive Th1 immunity and confer significant protection against early pulmonary TB, supporting further exploration of SapM as a vaccine antigen and AAV-based mucosal gene vaccination as a platform for TB vaccine development.

## 1. Introduction

Tuberculosis (TB) remains one of the leading causes of infectious disease-related mortality worldwide. According to recent estimates from the World Health Organization, there were approximately 10.7 million new TB cases and about 1.23 million TB-related deaths globally in 2024 [1]. Bacille Calmette–Guérin (BCG), the only licensed TB vaccine, is primarily used to help prevent severe forms of TB in young children, including miliary tuberculosis and tuberculous meningitis [2]. However, it shows highly variable and generally limited efficacy against adult pulmonary disease, particularly in preventing primary infection in the lungs and reactivation of latent infection [3,4,5,6]. These limitations highlight a critical gap in current TB vaccination strategies, especially for the control of transmission driven by adult pulmonary TB [5].

In this context, increasing attention has been focused on vaccine approaches capable of inducing robust mucosal immunity in the respiratory tract. Because *Mycobacterium tuberculosis* (*Mtb*) is transmitted via aerosols and establishes infection primarily within the lungs, while early immune responses at the site of pathogen entry are crucial for effective containment [7,8]. Local immune mechanisms in the lungs, including tissue-resident memory T cells, play a central role in rapid recognition and elimination of infected cells before bacterial dissemination [9,10]. Accordingly, vaccines that elicit durable, lung-local cellular immunity are considered particularly promising for improving protection against pulmonary TB.

Adeno-associated virus (AAV) vectors have attracted increasing attention as platforms for in vivo gene therapy and viral vectored vaccines, owing to their high gene transfer efficiency, capacity for sustained transgene expression, and relatively mild intrinsic immunogenicity [11,12]. Compared with conventional protein subunit vaccines or nucleic acid vaccines that drive only transient antigen expression, AAV vectors enable prolonged antigen production at the target site, thereby offering the sustained stimulation required for the induction and maintenance of memory T-cell responses [13,14]. When delivered via the respiratory tract, AAV-based vaccines can achieve high and persistent antigen expression directly within the lungs, efficiently engaging respiratory mucosal immunity and key effector populations, including lung tissue-resident T cells [15,16,17]. Consistent with this concept, evidence from animal models and early-phase clinical studies indicates that intranasal or inhaled administration of viral vectored vaccines enhances local immune responses in the lungs and improves protection against respiratory pathogens [18,19,20], supporting the development of AAV-based mucosal vaccination strategies [21,22].

Beyond vaccine platforms, antigen selection is equally critical. Pathogen-derived proteins that modulate host antimicrobial pathways represent an attractive and widely studied class of vaccine antigens, although their rational integration into effective vaccination strategies remains incomplete [23,24]. Immune responses targeting such proteins may enhance antigen-specific T-cell recognition of infected cells while alleviating pathogen-driven immune evasion mechanisms. Rv3310, encoded by the SapM gene, exemplifies this class of functional antigens. SapM dephosphorylates phosphatidylinositol 3-phosphate (PI3P) on the phagosomal membrane, thereby preventing acquisition of lysosomal components, blocking phagosome maturation and inhibiting phagosome–lysosome fusion [25,26,27]. In addition, SapM interferes with autophagy-related signaling pathways and suppresses autophagic flux, collectively promoting long-term intracellular survival of *Mtb* [28,29,30,31,32]. Consistent with these functions, SapM has been recognized as a key mediator of immune evasion and intracellular persistence, highlighting its relevance not only as a therapeutic target but also as a potential vaccine antigen [28,32].

Building on this rationale, we codon-optimized the *Mtb* SapM gene and expressed it using an adeno-associated virus (AAV) vector. Mice were immunized intranasally with recombinant AAV-SapM, and protective efficacy was evaluated in an aerosol *Mtb* infection model, together with analyses of vaccine-induced cellular immune responses. By integrating bacterial burden with immunological profiling, this study aims to provide proof-of-concept evidence supporting SapM as a feasible novel TB vaccine antigen and to establish experimental support for AAV-based mucosal vaccination strategies.

## 2. Materials and Methods

### 2.1. Cells and Bacterial Strains

HEK293T cells (ATCC) were maintained in Dulbecco’s modified Eagle medium (DMEM) supplemented with 10% fetal bovine serum (FBS) and 1% penicillin–streptomycin. Cells were cultured as adherent monolayers at 37 °C in a humidified incubator with 5% CO_2_, with medium changed routinely, and passaged when they reached approximately 80% confluence. The *Mycobacterium tuberculosis* H37Rv and BCG China strains were purchased from Shanghai Jingnuo Company (Shanghai, China) and cultured at 37 °C. The *Mtb* strains, including the virulent H37Rv and BCG strains, were grown at 37 °C. For liquid culture, strains were propagated in Middlebrook 7H9 broth (Becton Dickinson, Franklin Lakes, NJ, USA) supplemented with 0.5% glycerol, 0.25% Tween-80 and 10% oleic acid–albumin-dextrose–catalase (OADC, Becton Dickinson, Franklin Lakes, NJ, USA). For solid culture, bacteria were plated on Middlebrook 7H10 agar (Becton Dickinson, Franklin Lakes, NJ, USA) containing 0.5% glycerol and 10% OADC.

### 2.2. Construction of Recombinant pAAV Plasmids

The coding sequence of *Mtb* Ag85b and SapM was codon-optimized for expression in HEK293T cells and synthesized commercially. The optimized fragment was cloned into the pAAV-vector backbone (AAV plasmid, pAAV) to generate the recombinant plasmid pAAV-Ag85b and pAAV-SapM. The resulting construct was transformed into *E.coli* stbl3-competent cells, and single colonies were picked for screening. Positive clones were identified by colony PCR and further verified by Sanger sequencing to confirm the integrity of the insert and the correct reading frame. Verified clones were then expanded, and high-quality plasmid DNA was prepared using an EndoFree Plasmid Maxi Kit (Qiagen, Hilden, Germany). Helper plasmid pHelper and packaging plasmid pAAV-RC6 were transformed and prepared in an analogous manner.

### 2.3. Production of Recombinant rAAV-Ag85b and rAAV-SapM

Recombinant AAV-Ag85b and AAV-SapM were produced in HEK293T cells using a conventional triple-plasmid transfection approach. Briefly, HEK293T cells were seeded in culture dishes and transfected at near-confluent density with pAAV-Ag85b or pAAV-SapM, packaging plasmid pAAV-RC6 or pAAV-RC9 and the helper plasmid at an equimolar ratio using Lipofectamine 3000 (Thermo Fisher Scientific, Waltham, MA, USA) according to the manufacturer’s instructions. After 72 h of culture post transfection, cells were harvested and lysed, and crude viral lysates were clarified by low-speed centrifugation to remove cellular debris. The clarified supernatant was further purified by iodixanol density-gradient ultracentrifugation: centrifugation was performed at 4 °C and 200,000× *g* for 2 h, and 40% of the viral components in the zone were collected. The fraction corresponding to the rAAV6-Ag85b, rAAV9-Ag85b and rAAV6-SapM peaks was collected and subjected to buffer exchange in sterile phosphate-buffered saline (PBS) to remove iodixanol and residual small-molecule contaminants. The resulting preparation was aliquoted and stored for subsequent in vivo and in vitro experiments.

### 2.4. Assessment of rAAV Purity and Vector Genome Titers

The purity of rAAV preparations was evaluated by SDS–PAGE, followed by silver staining. Briefly, aliquots of purified viral preparations were mixed with SDS sample buffer, denatured by heat, and resolved on SDS–PAGE gels. After electrophoresis, gels were processed using a commercial silver staining kit according to the manufacturer’s instructions. The presence of the three characteristic AAV capsid proteins (VP1, VP2 and VP3) with minimal background bands was used as an indicator of preparation purity.

Vector genome titers were determined by quantitative PCR (qPCR). Viral samples were treated with DNase to remove contaminating free DNA and subsequently heat-inactivated. qPCR was performed using primers specific for the AAV vector genome (forward: 5′-TCGCTCACTGAGGCCG-3′; reverse: 5′-GGAACCCCTAGTGATGGAGTT-3′), and a standard curve was generated from serial dilutions of a plasmid containing the pAAV-SapM construct at known copy numbers. Vector genome titers were calculated from the standard curve and expressed as viral genomes per milliliter (vg/mL).

### 2.5. Western Blot

The expression of pAAV-Ag85b and pAAV-SapM in HEK293T cells was detected by Western blotting. HEK293T cells were infected with pAAV-Ag85b and pAAV-SapM, and cells were harvested 48 h post infection. Lysis was performed using RIPA buffer supplemented with PMSF. After mixing with loading buffer, the mixture was boiled at 100 °C for 10 min and centrifuged at 12,000 rpm for 5 min, after which the supernatants were loaded onto 10% SDS-PAGE gels for protein separation. Following electrophoresis, proteins were transferred to 0.45 μm PVDF membranes, which were then blocked with 5% BSA and sequentially incubated with a primary anti-Flag antibody and an HRP-conjugated goat anti-mouse IgG secondary antibody, with washing steps performed according to the manufacturers’ instructions. Signal detection was carried out using an enhanced chemiluminescence (ECL) substrate (Millipore, Burlington, MA, USA, WBKLS0100), and chemiluminescent bands were visualized and recorded using a Clinx ChemiDox imaging system (Bio-Rad, Hercules, CA, USA).

### 2.6. Generation of BMDCs and rAAV Infection

Bone marrow cells were harvested from femurs of 6-week-old SPF female C57BL/6J mice under sterile conditions after removal of the epiphyses. Cells were collected by centrifugation, resuspended in RPMI 1640 supplemented with GM-CSF, IL-4, and 10% FBS, then seeded into 10 cm culture dishes. After 24 h, half of the medium was replaced, and a second half-medium change was performed on day 4. On days 6–8 of differentiation, culture supernatants and adherent cells were collected together and centrifuged at 1000 rpm for 5 min. The cell pellet was resuspended in fresh medium and counted. BMDCs were then infected with rAAV at an MOI of 1 × 10^5^. As a positive control for DC maturation, cells were stimulated with 100 ng/mL of lipopolysaccharide (LPS) for 24 h. Infection was carried out in half the final culture volume for 2 h, after which an equal volume of fresh medium was added. The medium was replaced 24 h post infection, and cells were further cultured until 72 h. The proportion of EGFP-positive cells and the expression of maturation-associated surface markers (CD80, CD86, and MHC II) were analyzed by flow cytometry.

### 2.7. Mice and Immunization

Six-week-old specific pathogen-free (SPF) female C57BL/6J mice were purchased from Guangdong Vital River Laboratory Animal Technology Co., Ltd., and housed in the SPF-grade animal facility of Shenzhen University. All animal experiments were approved by the institution’s Animal Care and Use Committee and conducted under its supervision.

For immunization, an rAAV6-SapM, rAAV6-Ag85b or control rAAV6 vector was diluted to 2 × 10^11^ vg in 50 μL and administered intranasally to C57BL/6J mice (n = 12 per group). An intranasal PBS group (50 μL) was included as an infection-only control (n = 12). In the BCG group, mice received 1 × 10^6^ CFU BCG by subcutaneous injection as a positive control (n = 12). Six weeks after immunization, spleens were collected from a subset of mice (n = 6 per group) for immunogenicity assessment, while the remaining mice (n = 6 per group) were infected with *Mtb* H37Rv aerosols. Mice were monitored daily for general health status, including appearance, activity, and food/water intake. Body weight was recorded weekly from the day of immunization until the pre-challenge time point. To evaluate local tolerability prior to *Mtb* challenge, lungs were collected from a subset of mice 6 weeks post immunization and processed for H&E staining.

### 2.8. H37Rv Infection, Bacterial Quantification and Pathology

Six weeks after immunization, mice in each group (n = 6) were challenged with *Mtb* H37Rv (~100 CFU) using an aerosol exposure system (Glas-Col, Terre Haute, IN, USA). Four weeks post challenge, mice were euthanized under aseptic conditions, and the lungs and spleens were collected. The right upper lung lobe was fixed in 4% paraformaldehyde for subsequent histological analysis. The remaining lung tissue and the spleen were homogenized to generate single-organ suspensions, which were then subjected to serial dilution and plated onto Middlebrook 7H10 agar. Plates were incubated at 37 °C for 21 days, after which colony-forming units (CFUs) were counted to determine organ bacterial burden.

For histopathological assessment, lung lobes fixed for 24 h were processed by routine dehydration and paraffin embedding and sectioned at a thickness of 5 μm. Sections were stained with hematoxylin and eosin (H&E) and examined using a Leica DM microscope (Leica Microsystems, North Ryde, Australia) at 40× magnification. The degree of pulmonary inflammation in each section was evaluated semi-quantitatively using Image Viewer software. Whole lung slides were manually annotated in QuPath, and nucleated cells were detected using the built-in cell detection algorithm. A trained machine learning classifier was then applied to all sections to classify regions as airways, blood vessels, healthy parenchyma, or inflamed/immune-infiltrated tissue. Inflammation was quantified as the percentage of inflamed area relative to the total lung tissue area.

### 2.9. Cell Isolation, H37Rv Lysate Stimulation, and Flow Cytometry

Six weeks after immunization, lungs and spleens were aseptically harvested from mice in each group. Spleens were mechanically dissociated to obtain single-cell suspensions. Lungs were minced into small pieces and enzymatically digested with collagenase IV (Sigma-Aldrich) and DNase I (10 U/mL) for 30 min at 37 °C, followed by gentle mechanical disruption to generate single-cell suspensions. Red blood cells were removed by incubation with 1×RBC lysis buffer for 10 min at room temperature, followed by centrifugation and washing.

For lung immune phenotyping, lung single-cell suspensions were subjected to surface staining without ex vivo antigen stimulation using antibodies against CD45, CD3, CD4, CD8, CD69, CD103, CD44, and CD62L. For splenocyte functional assays, splenocytes were resuspended, adjusted to 1 × 10^6^ cells/mL, and plated in tissue culture plates. H37Rv whole-cell lysate was prepared from mid-log-phase *Mtb* H37Rv cultures. After harvesting, bacteria were washed with PBS and heat-inactivated at 80 °C for 30 min, followed by lysis on ice by sonication. Insoluble debris was removed by centrifugation, and the protein concentration of the clarified supernatant was determined using a BCA assay. For intracellular cytokine staining (ICS), H37Rv lysate was added to a final concentration of 5 μg/mL, and cultures were incubated for 4 h at 37 °C in a humidified 5% CO_2_ incubator. Monensin (GolgiStop) and brefeldin A (GolgiPlug) were subsequently added, and cells were incubated overnight to inhibit cytokine secretion and promote intracellular cytokine accumulation. For ELISA experiments, after adding H37Rv lysate, the cells were directly incubated in a 37 °C, 5% CO_2_ incubator for 72 h, and the cell supernatant was collected for IFN-γ secretion-level detection.

After stimulation, cells were prepared for flow cytometry following the manufacturers’ instructions. Briefly, cells were first stained with a fixable viability dye (BioLegend, San Diego, CA, USA) to exclude dead cells, then incubated with fluorochrome-conjugated antibodies against surface markers. For lung surface phenotyping, samples were fixed without permeabilization. For splenocyte ICS, cells were fixed and permeabilized using Fix/Perm solution (BD Biosciences, San Jose, CA, USA) for 30 min at 4 °C in the dark and subsequently stained with cytokine-specific antibodies for intracellular detection. Samples were acquired on a Beckman Coulter flow cytometer, and data were analyzed using FlowJo (v10.8.1) to quantify the frequencies of defined immune-cell subsets and cytokine-positive populations. For ELISA, cell supernatant was collected after 72 h, and detection was performed according to the Mouse IFN-γ ELISA kit instructions (Hangzhou Youke, Hangzhou, China), using a microplate reader set to 450 nm corrected with 570 nm.

### 2.10. Data Analysis

All statistical analyses were performed using GraphPad Prism 10 (GraphPad Software, version 8.0.2, La Jolla, CA, USA). Comparisons among multiple groups were conducted using one-way analysis of variance (one-way ANOVA). Statistical significance was defined as * *p* < 0.05, ** *p* < 0.01, *** *p* < 0.001, and **** *p* < 0.0001.

## 3. Results

### 3.1. Establishment and Optimization of an rAAV Packaging and Purification Platform for Intranasal TB Vaccine Candidates

**Figure 1:**
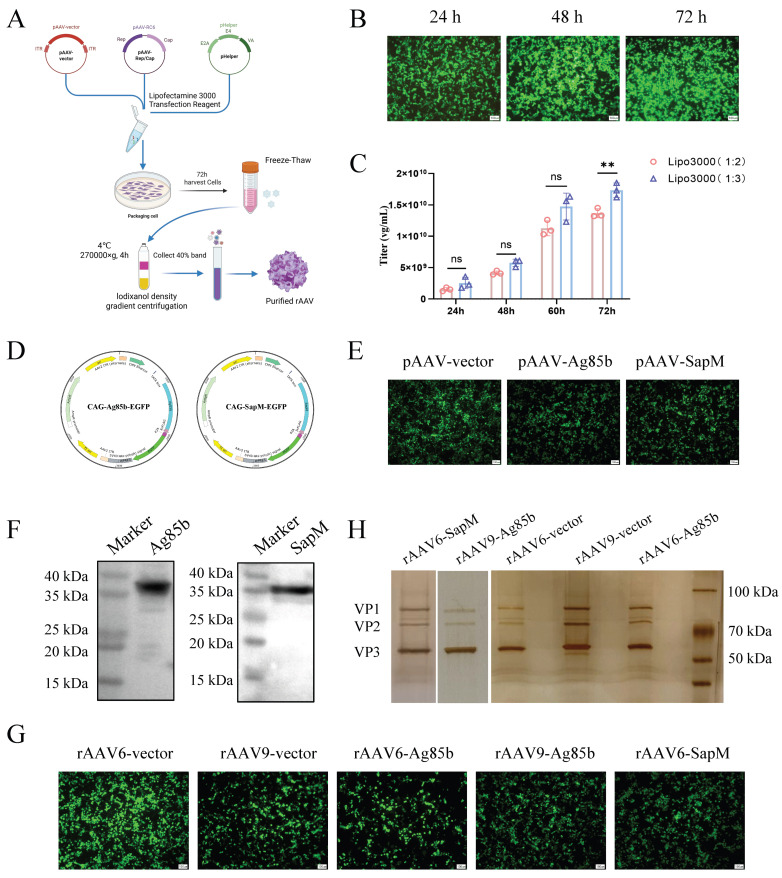
**Construction and verification of the recombinant AAV-based vaccine candidates.** (**A**) Schematic overview of the rAAV packaging and purification workflow created with BioRender.com. (**B**) Representative fluorescence images of HEK293T cells following triple-plasmid transfection for rAAV6-vector packaging, acquired 24, 48, and 72 h post transfection (hpt). (**C**) Viral genome (vg) titers of the rAAV6 vector harvested 24 h, 48 h, 60 h, and 72 hpt, quantified by quantitative PCR (qPCR). (**D**) Maps of recombinant plasmids pAAV-Ag85b (left) and pAAV-SapM (right). (**E**) Representative EGFP fluorescence in HEK293T cells 48 h after transfection with pAAV-Ag85b or pAAV-SapM. (**F**) Ag85b (~37 kDa) and SapM (~35 kDa) expression in HEK293T cells detected by Western blotting using an anti-3×FLAG antibody. (**G**) Representative EGFP fluorescence 24 h after triple-plasmid co-transfection for rAAV packaging. (**H**) Iodixanol gradient purification followed by silver staining of capsid proteins showing VP1 (~87 kDa), VP2 (~72 kDa), and VP3 (~62 kDa). Data are shown as mean ± SEM (n = 3). Statistical significance was determined by multiple *t*-tests. ns, not significant; ** *p* < 0.01.

We established a reproducible rAAV packaging and purification workflow suitable for the generation of intranasal TB vaccine candidates and successfully constructed rAAV-Ag85b and rAAV6-SapM vectors. To optimize rAAV production, the rAAV6 vector was packaged in HEK293T cells using a triple-plasmid transfection with a DNA-to-transfection reagent ratio of 1:2 or 1:3. Cells were harvested 24 h, 48 h, 60 h, and 72 h post transfection, and vector genome titers were determined from crude viral lysates. The highest rAAV titers were consistently obtained using a 1:3 transfection ratio with cell harvest 72 h post transfection (Figure 1A–C), and this condition was selected for subsequent vector production.

To generate candidate TB vaccine vectors, the Ag85b and SapM coding sequences were codon-optimized for mammalian expression and cloned into a pAAV backbone. Ag85b was included as a canonical protective antigen extensively used in TB vaccine studies [33,34,35], providing a benchmark for evaluating rAAV vector production, transgene expression, and serotype suitability. Correct insertion, reading-frame integrity, and sequence fidelity were confirmed by restriction analysis and Sanger sequencing, indicating successful construction of pAAV-Ag85b and pAAV-SapM (Figure 1D). Transient transfection of HEK293T cells with the recombinant plasmids resulted in robust transgene expression, as indicated by EGFP fluorescence and confirmed by Western blot analysis, which detected protein bands of the expected molecular weights for both Ag85b and SapM (Figure 1E,F and Figure S1).

Because both AAV6 and AAV9 serotypes have been reported to efficiently transduce the nasal mucosa and airway epithelial cells [36,37,38], we next compared their suitability for intranasal TB vaccination. rAAV6-Ag85b and rAAV9-Ag85b vectors were produced using the optimized workflow and purified by iodixanol density-gradient ultracentrifugation. Silver staining of purified preparations revealed the characteristic AAV capsid proteins VP1, VP2, and VP3 with minimal background, indicating high vector purity (Figure 1G,H and Figure S2). Vector genome titers quantified by qPCR reached levels sufficient for in vivo immunization at 2.0 × 10^11^ vg per mouse. Collectively, these results demonstrate the successful establishment of a high-yield and high-purity rAAV production and purification platform, enabling the production of reliable rAAV6-Ag85b, rAAV9-Ag85b, and rAAV6-SapM for subsequent intranasal immunization studies.

### 3.2. AAV6 Efficiently Transduces Antigen-Presenting Cells Without Inducing Phenotypic Activation

**Figure 2:**
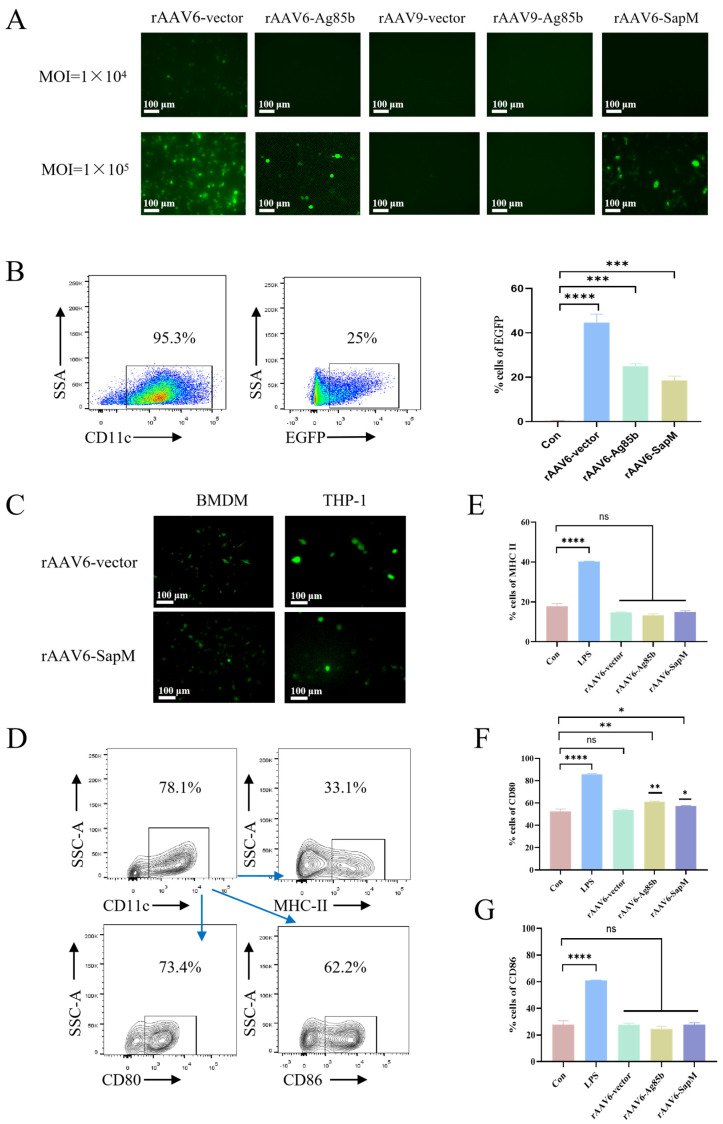
**Serotype screening identifies AAV6 as an optimal vector for APC transduction.** (**A**) Immature BMDCs were infected with rAAV6-Ag85b, rAAV9-Ag85b, or rAAV6-SapM at a multiplicity of infection (MOIs) of 1 × 10^4^ or 1 × 10^5^. Representative EGFP fluorescence images were acquired 72 h post infection (hpi). (**B**) Transduction efficiency in BMDCs infected with rAAV6-Ag85b or rAAV6-SapM (MOI = 1 × 10^5^) was quantified by flow cytometry and expressed as the percentage of EGFP-positive cells. (**C**) BMDMs and THP-1 cells were infected with the rAAV6 vector or rAAV6-SapM at an MOI of 1 × 10^5^, and representative EGFP fluorescence images were acquired 72 hpi. (**D**) Flow cytometry gate strategy. (**E**–**G**) BMDCs were infected with rAAV6-Ag85b or rAAV6-SapM (MOI = 1 × 10^5^) for 72 h. LPS was used as a positive control for DC maturation. The percentages of cells positive for maturation-associated markers were quantified by flow cytometry. (**E**) MHC II. (**F**) CD86. (**G**) CD80. Data are shown as mean ± SEM (n = 3). Statistical significance was determined by one-way ANOVA. ns, not significant; * *p*< 0.05, ** *p*< 0.01; *** *p* < 0.001; **** *p* < 0.0001.

To identify the optimal AAV serotype for antigen-presenting cell (APC) targeting, we compared AAV6 and AAV9 for their ability to transduce dendritic cells and macrophages. Immature bone marrow-derived dendritic cells (BMDCs) were infected with rAAV6-Ag85b or rAAV9-Ag85b at an MOI of 1 × 10^4^ or 1 × 10^5^. At 72 h post infection, robust EGFP fluorescence was readily detected in the rAAV6-Ag85b group, with transduction efficiency increasing in a dose-dependent manner. In contrast, rAAV9-Ag85b showed negligible transduction of immature BMDCs under the same conditions (Figure 2A). To determine whether AAV6 could also transduce macrophages, bone marrow-derived macrophages (BMDMs) and THP-1-derived macrophages were infected with the rAAV6 vector at an MOI of 1 × 10^5^. These results demonstrated that AAV6 not only efficiently transduces BMDCs but is also capable of transducing macrophages (Figure 2C).

We next assessed whether AAV6-mediated transduction alters the phenotypic maturation status of DCs. Immature BMDCs were infected with rAAV6-Ag85b or rAAV6-SapM at an MOI of 1 × 10^5^, and transgene expression was evaluated 72 h post infection by fluorescence microscopy and flow cytometry. The frequencies of EGFP-positive cells were 42.2%, 25.0%, and 17.1% in the rAAV6-vector, rAAV6-Ag85b, and rAAV6-SapM groups, respectively, compared with 0.5% in the uninfected control (Figure 2B). LPS was treated as a positive control for DC maturation. Importantly, the proportions of CD86- and MHC II-positive cells were comparable between the rAAV6-Ag85b/rAAV6-SapM groups and the uninfected control, with only a modest increase observed in the percentage of CD80 expression, suggesting that AAV6 transduction does not result in appreciable DC maturation or activation (Figure 2D–G). Based on these findings, AAV6 was selected as the preferred serotype for the generation of SapM-expressing AAV vectors in subsequent experiments.

### 3.3. Intranasal Immunization with rAAV6-SapM Elicited Antigen-Specific Th1-Biased Cellular Immune Responses Characterized by Enhanced IFN-γ Production

**Figure 3:**
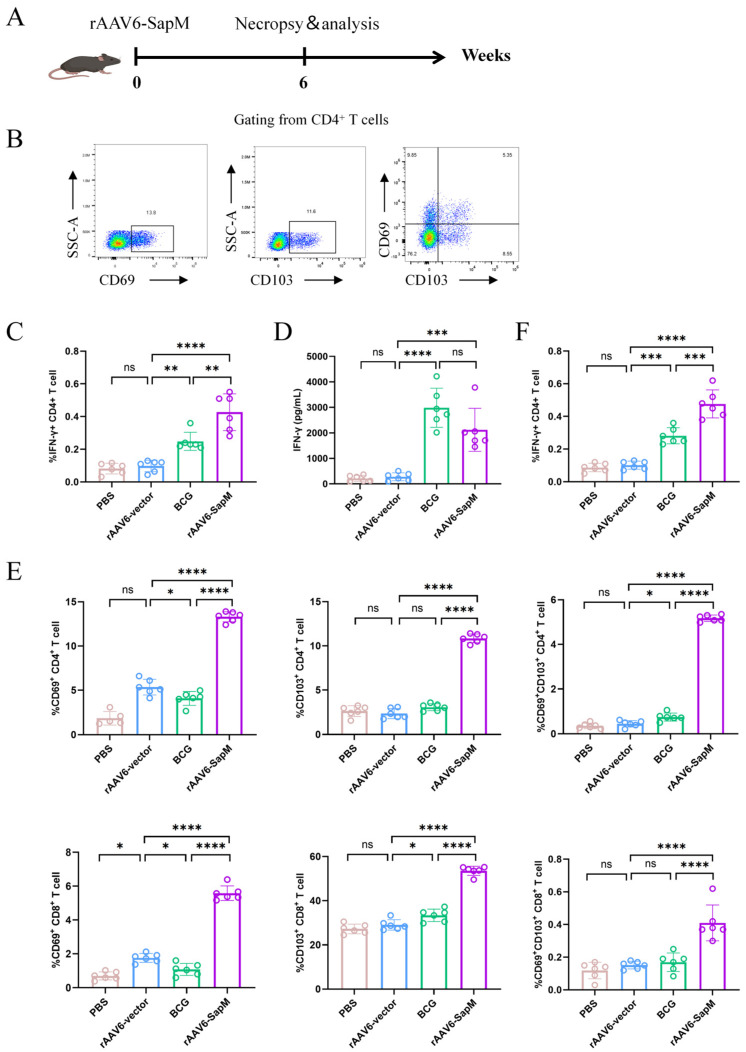
**A single intranasal dose of rAAV6-SapM vaccine elicited a robust Th1-biased immune response.** (**A**) Immunization scheme. Female C57BL/6 mice (n = 6 per group) were vaccinated subcutaneously (s.c.) with BCG (1 × 10^6^ CFU) or intranasally (i.n.) with rAAV6-SapM (2 × 10^11^ vg). Control mice received the same dose of the rAAV6 vector. (**B**) Gating strategy for lung CD4^+^ TRM-like phenotyping. After gating live singlets, lung leukocytes were identified as CD45^+^ cells, and T cells were identified as CD3^+^ cells. CD4^+^ T cells were then gated within CD3^+^ cells, and the frequencies of CD69^+^, CD103^+^, and CD69^+^CD103^+^ (double-positive) subsets were quantified. Values denote percentages in each gate. (CD8^+^ gating was performed similarly.) (**C**) At 6 weeks post immunization, splenocytes were stimulated ex vivo with H37Rv lysate, and the frequency of IFN-γ-producing CD4^+^ T cells was quantified by intracellular cytokine staining (ICS). (**D**) IFN-γ levels in culture supernatants following ex vivo stimulation were measured by ELISA. (**E**) Perfused lung tissue was harvested at week 6 post immunization, and lung single-cell suspensions were analyzed by flow cytometry. Frequencies of CD69^+^, CD103^+^, and CD69^+^CD103^+^ (double-positive) subsets among lung CD4^+^ (top row) and CD8^+^ (bottom row) T cells. (**F**) Lung cells were stimulated ex vivo with H37Rv lysate, and the frequency of IFN-γ-producing CD4^+^ T cells was quantified by ICS. Data are plotted as mean ± SEM (n = 6). Group differences were analyzed using one-way ANOVA. ns, not significant; * *p*< 0.05, ** *p*< 0.01; *** *p* < 0.001; **** *p* < 0.0001.

To characterize antigen-specific cellular immunity induced by rAAV6-SapM, splenocytes were harvested six weeks after immunization, prior to H37Rv challenge, and stimulated in vitro with H37Rv lysate (Figure 3A, Figure S3A). Antigen-specific IFN-γ responses were assessed by intracellular cytokine staining and ELISA. Compared with the rAAV6-vector control group, splenocytes from rAAV6-SapM-immunized mice exhibited a significantly increased frequency of IFN-γ^+^ CD4^+^ T cells following H37Rv lysate stimulation, indicating the induction of a Th1-biased, *Mtb* antigen-responsive cellular immune profile (Figure 3B,C). Consistently, ELISA analysis showed markedly elevated IFN-γ secretion in the rAAV6-SapM group, reaching approximately 2000 pg/mL, compared with rAAV6 vector-immunized controls (Figure 3D). These data demonstrate that intranasal rAAV6-SapM immunization effectively primes antigen-responsive Th1-type cellular immunity. In addition, we also evaluated the rAAV6-Ag85b immunization group. The rAAV6-SapM group showed a higher frequency of IFN-γ^+^ CD4^+^ T cells than the rAAV6-Ag85b group, whereas no obvious differences were observed in IL-2^+^ or TNF-α^+^ CD4^+^ T cells between the two groups (Figure S4A).

Additionally, 6 weeks post immunization, mice were perfused and lungs were analyzed by flow cytometry to characterize TRM-like phenotypes (Figure S3B). Compared with the rAAV6-vector control and BCG groups, intranasal rAAV6-SapM vaccination significantly increased the frequency of CD69^+^ cells among pulmonary CD4^+^ and CD8^+^ T cells and enhanced CD103 expression. Further analysis revealed a significant expansion of the CD69^+^CD103^+^ double-positive population in the lungs, indicating that intranasal immunization more efficiently establishes tissue-associated/tissue resident-like T-cell phenotypes at the respiratory interface (Figure 3B,E). In addition to phenotypic profiling, we assessed pulmonary CD4^+^ T-cell IFN-γ responses. Following ex vivo stimulation with H37Rv lysate, the frequency of IFN-γ^+^ CD4^+^ T cells in the lungs was significantly higher in the rAAV6-SapM group than in the rAAV6-vector group and also exceeded that in the BCG group, suggesting that intranasal rAAV6-SapM elicits stronger local Th1-like functional responses in the lungs (Figure 3F).

Throughout the study, all mice remained in good general condition, with food intake and activity levels comparable to those of naive mice, and no deaths or obvious clinical abnormalities were observed. Bodyweight monitoring showed that body weights remained generally stable over time in all groups, with no significant differences among groups. Furthermore, H&E staining of lung tissues collected 6 weeks post immunization revealed no overt pathological changes, such as inflammatory infiltrates, hemorrhage, or tissue necrosis, indicating that intranasal delivery of rAAV6-SapM was well tolerated and demonstrated preliminary safety in C57BL/6J mice (Figure S5).

### 3.4. Intranasal Vaccination with rAAV6-SapM Conferred Significant Protection Against Aerosol H37Rv Challenge in Mice

**Figure 4:**
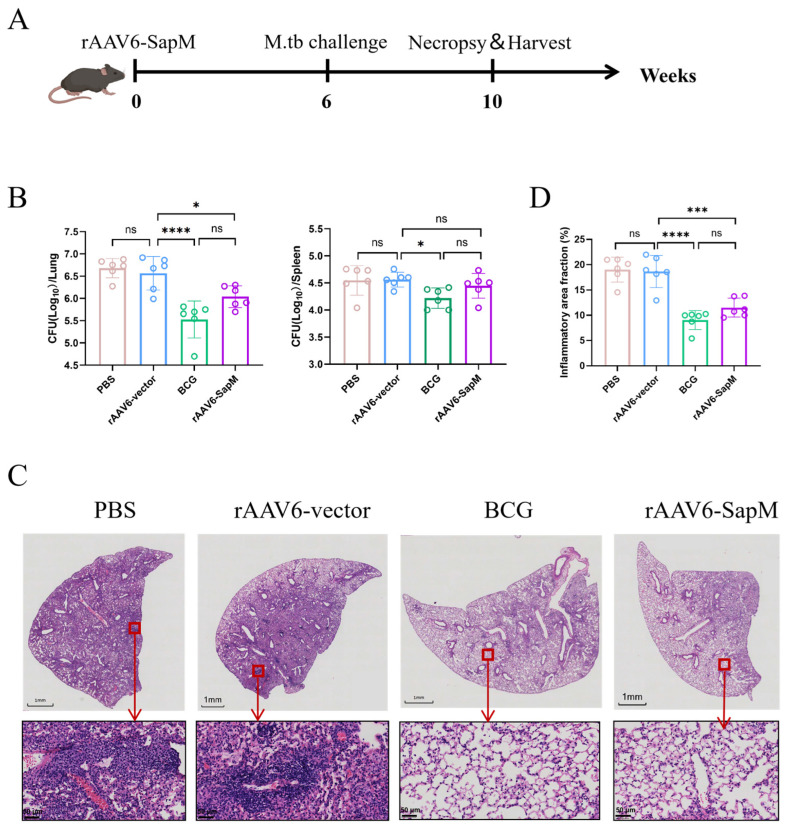
**Protective efficacy of rAAV6-SapM vaccine against aerosol *Mtb* (H37Rv) in the C57BL/6J model.** (**A**) Experimental schematic of immunization and aerosol challenge. (**B**) Bacterial burdens in the lungs and spleen 4 weeks post challenge. (**C**) Representative lung H&E sections 4 weeks post H37Rv challenge. Scale bars equal 1mm. Outlined areas in the main images indicate enlarged regions, with a scale bar of 50 μm. (**D**) Quantification of lung inflammation was performed on whole-slide images, calculated as the percentage of the inflamed area relative to the total lung tissue area using Qupath software (V0.5.1). Data are plotted as mean ± SEM (n = 6). Group differences were analyzed using one-way ANOVA. ns, not significant; * *p* < 0.05, *** *p* < 0.001; **** *p* < 0.0001.

To systematically evaluate the protective efficacy of rAAV6-SapM, mice were challenged with aerosolized H37Rv six weeks after a single intranasal immunization, and bacterial burdens in the lungs and spleens were quantified four weeks post challenge by CFU enumeration (Figure 4A). Compared with the rAAV6 vector and PBS control group, intranasal immunization with rAAV6-SapM resulted in a significant reduction in pulmonary CFUs (Figure 4B). Although the level of protection was lower than that achieved by the BCG intramuscular vaccination, the statistically significant reduction relative to the empty-vector control indicates that rAAV6-SapM confers meaningful antibacterial activity during the early phase of primary pulmonary infection. rAAV6-Ag85b immunization also significantly reduced lung CFUs, with no significant difference compared with rAAV6-SapM (Figure S4B).

Histopathological findings were consistent with the CFU data. Hematoxylin and eosin (HE) staining showed that the lungs of the PBS and rAAV6-vector groups exhibited widespread inflammatory involvement, characterized by dense interstitial inflammatory cell infiltration, thickening of the alveolar septa, and partial disruption of alveolar spaces. In contrast, lungs from the BCG and rAAV6-SapM groups displayed reduced interstitial thickening, smaller and more discrete inflammatory foci, better preservation of alveolar architecture, and less parenchymal damage (Figure 4C). Consistent with these histological differences, whole-slide quantitative inflammation scoring by QuPath demonstrated a significantly lower inflammatory burden in the BCG and rAAV6-SapM groups compared with the PBS and rAAV6-vector control groups (Figure 4D). In addition, the pathological severity was comparable between the rAAV6-SapM and rAAV6-Ag85b groups (Figure S4C,D). These pathological improvements aligned with the reduced bacterial burden, supporting a protective effect of rAAV6-SapM against infection-associated pulmonary inflammation and injury. In the spleen, the rAAV6-SapM group showed a trend toward fewer CFUs compared with rAAV6-vector controls, although this difference did not reach statistical significance (Figure 4B). Overall, intranasal rAAV6-SapM immunization effectively reduced pulmonary mycobacterial burden and mitigated lung pathology to a measurable extent.

## 4. Discussion

In this study, we developed and optimized a reproducible rAAV production and purification platform suitable for mucosal immunization and applied it to deliver *Mtb* antigen SapM via the intranasal route. Using this strategy, we demonstrated that a single intranasal administration of rAAV6-SapM induces a Th1-biased cellular immune response characterized by enhanced IFN-γ production and confers partial but significant protection against aerosol *Mtb* challenge, as reflected by reduced pulmonary bacterial burden and attenuated lung pathology. Together, these findings provide proof-of-concept support for SapM as a feasible TB vaccine antigen and highlight the potential of AAV-based mucosal delivery platforms for tuberculosis vaccination.

Given that *Mtb* primarily enters through the respiratory tract and establishes infection in the lungs, intranasal immunization is considered to provide a more direct immune response at the site of pathogen invasion, offering faster local control in the early stages of infection. To date, multiple technical pathways have been explored for mucosal tuberculosis vaccines. Mucosal BCG has demonstrated strong local pulmonary immune induction capabilities in various animal models, and its protective effect is superior to that of subcutaneous injection [39]. Meanwhile, various viral vectors (such as adenovirus and MVA) can enhance local mucosal-cell immunity after respiratory delivery. For example, nebulized MVA85A has been shown to be well tolerated in healthy BCG-vaccinated adults and can induce antigen-specific T-cell responses at mucosal sites such as in bronchoalveolar lavage fluid [40]. However, the variability of prior immunity, persistence of immunity, and cross-model protective effects of different vectors remain concerns. In addition to vector strategies, intranasal administration of Ag85b-ESAT-6 combined with mucosal adjuvants can also enhance protection against *Mtb* infection and enhance prior BCG immunity [41]. Overall, these studies suggest that mucosal immunity has advantages in the development of novel tuberculosis vaccines while also indicating that different strategies have their own limitations in terms of persistence of immunity, safety, and reproducibility of protection. Based on this, we further explore rAAV6 in this paper. The immunological characteristics and protective effects of mucosal delivery platforms are examined to provide a clearer reference for their potential advantages and limitations within the aforementioned dimensions.

Genetic deletion of SapM, alone or in combination with other virulence-associated genes, results in attenuated bacterial growth and enhanced host immune activation in multiple animal models [42,43]. While these studies have focused on weakening the pathogen through removal of SapM, our work explores the same target from a complementary perspective by actively presenting SapM as a vaccine antigen. Despite the conceptual difference between gene deletion and antigen-based vaccination, both approaches converge on a consistent conclusion: SapM is a key determinant of bacterial persistence, and immune targeting of this pathway represents a rational strategy for intervention. On this basis, we prioritized SapM for antigen development for three immunological reasons. First, SapM is expressed during infection and contributes to intracellular survival by modulating host processes such as phagolysosome maturation and autophagy. As a secreted or host-exposed factor, SapM is more likely to be processed and presented by infected antigen-presenting cells, enabling SapM-specific T cells to recognize and eliminate infected cells [30,44]. Second, because SapM supports bacterial fitness and virulence, it is likely under a stronger evolutionary constraint than dispensable antigens, potentially lowering the risk of immune escape—an established rationale in subunit and viral vectored vaccine design [29,45]. Third, our antigen-selection strategy is consistent with established TB vaccine paradigms in which virulence-associated secreted effectors are repeatedly prioritized as immunogens. For example, ESAT-6 is an ESX-1-secreted antigen implicated in phagosome-associated host pathways and is incorporated into clinical subunit candidates such as H56:IC31 [46]. In parallel, TB10.4 is an ESX-3-secreted effector that can impair phagosome maturation by interfering with the ESCRT machinery and is included in candidates such as H4:IC31 [47]. Collectively, these precedents support SapM as an immunologically accessible, functionally important, and conceptually validated antigen target for vaccination.

The enhanced IFN-γ response observed in this study is highly consistent with the classic understanding of protective immunity against tuberculosis. Extensive evidence indicates that IFN-γ-centric Th1 responses are critical for controlling intracellular pathogens, and hosts lacking IFN-γ or related signaling pathways are highly susceptible to *Mtb* infection [48,49]. In our study, splenocytes from rAAV6-SapM-immunized mice produced significantly higher levels of IFN-γ upon stimulation with H37Rv lysate, which corresponded well to the decrease in lung CFUs, suggesting that vaccine-induced Th1 response likely contributed to bacterial control. Nevertheless, our immunological analysis was limited to bulk IFN-γ measurements and did not resolve antigen-specific T-cell subsets or tissue-resident populations. Future studies employing multiparameter flow cytometry or single-cell approaches will be required to delineate the precise cellular mechanisms underlying protection.

From the perspective of vaccine delivery platforms, this study used an AAV vector to deliver SapM via the intranasal route. Viral vector vaccines have been explored in tuberculosis research, with adenovirus-based vectors demonstrating enhanced pulmonary mucosal immunity and tissue-resident T-cell responses when administered via the respiratory tract, often providing superior pulmonary protection compared with intramuscular injection in various animal models [50,51,52]. Consistent with these observations, recent studies of inhaled or intranasally delivered viral vectored TB vaccines support the concept that airway mucosal immunity can outperform purely systemic immunity, particularly under aerosol challenge conditions [7,53].

In this context, AAV offers several attractive features, including comparatively mild intrinsic immunogenicity and the capacity for sustained transgene expression, which may support prolonged local antigen availability in the lungs. In our study, a single intranasal dose of rAAV6-SapM was sufficient to induce a measurable antigen-responsive IFN-γ signal at six weeks and to significantly reduce pulmonary bacterial burden. Importantly, we further observed an increased frequency of lung T cells expressing residency-associated markers CD69 and CD103, together with *Mtb* antigen-stimulated IFN-γ production by lung CD4^+^ T cells, supporting the induction of a lung-localized cellular immune response following mucosal vaccination. Although lung-resident T-cell subsets or tissue-resident memory populations were not directly assessed, these findings are consistent with the potential benefit of combining durable local antigen expression with mucosal vaccine delivery and warrant further mechanistic investigation of pulmonary immune responses.

Several limitations of this study should be acknowledged. First, although rAAV6-SapM vaccination yielded a statistically significant reduction in lung CFUs, the magnitude of protection was modest compared with BCG, indicating substantial room for improvement. Future studies will evaluate heterologous prime–boost immunization strategies, such as priming with BCG followed by boosting with rAAV6-SapM, to determine whether this regimen can further enhance Th1-biased immune responses and provide additional protection beyond BCG alone. Given the complex antigenic repertoire of and stage-specific biology of *Mtb*, a single antigen is unlikely to capture all immunologically relevant targets. Future work should therefore explore multivalent designs that combine SapM with well-established protective antigens (e.g., the Ag85 complex, CFP-10) and/or antigens associated with chronic infection or latency, leveraging the AAV platform to enable coordinated expression of multiple antigens and to assess potential gains in breadth and protective potency [8]. In addition, subcutaneous BCG was used as the only positive control regimen in this study. Future work will include mucosal BCG as a comparator to enable head-to-head evaluation of mucosal immunogenicity and protective efficacy at the respiratory interface. Second, bacterial burden was assessed at a single early time point following challenge, precluding conclusions regarding long-term control or disease progression. Extended follow-up studies will be necessary to evaluate durability of protection. Finally, while AAV vectors have demonstrated a favorable safety profile in clinical gene therapy, translation to TB-endemic settings will require careful consideration of pre-existing anti-AAV immunity and potential immunity-related effects.

In summary, our study demonstrated, in a mouse model of tuberculosis aerosol infection, that nasal delivery of a recombinant AAV vector expressing SapM can induce a significant IFN-γ-mediated cellular immune response and effectively reduce the pulmonary tuberculosis burden in the early stages of infection. Combined with previous research on the attenuation and enhanced immunogenicity of SapM-deleted strains, this study further supports the rationale for SapM as a target for tuberculosis vaccines and suggests that targeting virulence factors using an AAV mucosal gene vaccine platform is a strategy worthy of further exploration.

## 5. Conclusions

In this study, we established and optimized an rAAV production and purification platform suitable for mucosal immunization and constructed an AAV6-based vaccine expressing *Mtb* antigen SapM (rAAV6-SapM). A single intranasal dose of rAAV6-SapM induced mucosal T-cell responses in the lungs and increased CD69 and CD103 while also eliciting a robust Th1 cellular response characterized by enhanced IFN-γ production and conferring protection in an H37Rv aerosol challenge model, as evidenced by reduced pulmonary bacterial burden and attenuated lung pathology. These findings support SapM as a protective antigen candidate and demonstrate the feasibility of AAV vectors as a mucosal delivery platform for TB vaccination, providing a basis for the development of next-generation TB vaccines. Future studies will prioritize combination strategies integrating rAAV6-SapM with additional antigens and/or complementary vaccine platforms and will assess the durability of protection to more fully define the translational potential of AAV-based mucosal immunization.

## Data Availability

The original contributions presented in this study are contained within the article.

## Supplementary Materials

The following supporting information can be downloaded at: https://www.mdpi.com/article/10.3390/vaccines14030224/s1, Figure S1: Uncropped original Western blot images for Figure 1G. Representative uncropped Western blots of (A) Ag85b (~37 kDa) and (B) SapM (~35 kDa) expressed in HEK293T cells and probed with anti-3×FLAG. Red boxes indicate the bands presented in Figure 1G. Molecular weight markers (kDa) are indicated. Figure S2: Uncropped original silver-stained gels corresponding to Figure 1H. Representative uncropped silver staining of (A) rAAV6-SapM; (B) rAAV9-Ag85b; and (C) rAAV6-vector, rAAV9-vector and rAAV6-Ag85b. The three major capsid protein bands, from top to bottom, correspond to VP1 (~87 kDa), VP2 (~72 kDa), and VP3 (~62 kDa). Red boxes indicate the bands presented in Figure 1H. Molecular weight markers (kDa) are indicated. Figure S3. Flow cytometry gating strategies for splenocytes and lung cells. (A) Gating for cytokine production in splenocytes. At 6 weeks post immunization, splenocytes were stimulated ex vivo with H37Rv lysate. Lymphocytes were identified by FSC-A/SSC-A, followed by singlet discrimination and dead-cell exclusion. Within the live CD3^+^ T-cell population, CD4^+^ and CD8^+^ subsets were identified. The frequencies of IFN-γ-, TNF-α- and IL-2-producing cells were analyzed within the gated CD4^+^ T-cell population. (B) Gating for lung tissue-resident memory T cells. At 6 weeks post-immunization, lung mononuclear cells were analyzed without further stimulation. Following sequential gating of lymphocytes, singlets, and live cells, CD3^+^ T cells were identified within the CD45^+^ leukocyte population. T cells were further subdivided into CD4^+^ and CD8^+^ populations. The expression of resident markers CD69 and CD103 was analyzed within both CD4^+^ and CD8^+^ T-cell subsets (representative plots for CD4^+^ T-cells are shown; CD8^+^ gating was performed similarly). Numbers in the plots represent the percentage of cells within the indicated gates. Figure S4: Immunogenicity and protective efficacy of rAAV6-Ag85b. (A) At 6 weeks post immunization, splenocytes were stimulated ex vivo with H37Rv lysate, and the frequency of IFN-γ, TNF-α and IL-2 CD4+ T cells was quantified by ICS. (B) Bacterial burdens in the lungs and spleen 4 weeks post challenge. (C) Representative lung H&E sections 4 weeks post H37Rv challenge. Scale bars equal 1mm. Outlined areas in the main images indicate enlarged regions, with a scale bar of 50 μm. (D) Quantification of lung inflammation was performed on whole-slide images, calculated as the percentage of the inflamed area relative to the total lung-tissue area using Qupath software (V0.5.1). Data are plotted as mean ± SEM (n = 6). Group differences were analyzed using one-way ANOVA with Tukey’s test. ns, not significant; * *p* < 0.05, ** *p* < 0.01; *** *p* < 0.001; **** *p* < 0.0001. Figure S5: Safety assessment of rAAV6 immunization prior to *Mtb* challenge. (A) C57BL/6 mice were weighed weekly (Day 0–42) to monitor their weight gain before *Mtb* infection (n = 6). No significant differences in body weight were observed among groups (ns). (B) Representative H&E-stained lung sections collected 6 weeks after immunization (pre-challenge) from the indicated groups. No apparent increase in lung inflammation or tissue pathology was observed in rAAV6-immunized mice compared with PBS or vector controls. Group differences were analyzed using two-way repeated-measures ANOVA (group × time). ns, not significant.

## References

[1] World Health Organization (2025). Global Tuberculosis Report.

[2] Rodrigues L.C., Diwan V.K., Wheeler J.G. (1993). Protective effect of BCG against tuberculous meningitis and miliary tuberculosis: A meta-analysis. Int. J. Epidemiol..

[3] Trunz B.B., Fine P., Dye C. (2006). Effect of BCG vaccination on childhood tuberculous meningitis and miliary tuberculosis worldwide: A meta-analysis and assessment of cost-effectiveness. Lancet.

[4] Syggelou A., Spyridis N., Benetatou K., Kourkouni E., Kourlaba G., Tsagaraki M., Maritsi D., Eleftheriou I., Tsolia M. (2020). BCG Vaccine Protection against TB Infection among Children Older than 5 Years in Close Contact with an Infectious Adult TB Case. J. Clin. Med..

[5] Starshinova A., Kudryavtsev I., Rubinstein A., Dovgalyuk I., Kulpina A., Churilov L.P., Kudlay D. (2025). BCG vaccination: Historical role, modern applications, and future perspectives in tuberculosis and beyond. Front. Pediatr..

[6] Martinez L., Cords O., Liu Q., Acuna-Villaorduna C., Bonnet M., Fox G.J., Carvalho A.C.C., Chan P.C., Croda J., Hill P.C. (2022). Infant BCG vaccination and risk of pulmonary and extrapulmonary tuberculosis throughout the life course: A systematic review and individual participant data meta-analysis. Lancet Glob. Health.

[7] Li J., Wu G., Huang Z., Hu J., Tie X., Wu H., Wang Z., Chen K. (2025). Advances and prospects of respiratory mucosal vaccines: Mechanisms, technologies, and clinical applications. npj Vaccines.

[8] Lai R., Afkhami S., Haddadi S., Jeyanathan M., Xing Z. (2015). Mucosal immunity and novel tuberculosis vaccine strategies: Route of immunisation-determined T-cell homing to restricted lung mucosal compartments. Eur. Respir. Rev. Off. J. Eur. Respir. Soc..

[9] Yuan R., Yu J., Jiao Z., Li J., Wu F., Yan R., Huang X., Chen C. (2021). The Roles of Tissue-Resident Memory T Cells in Lung Diseases. Front. Immunol..

[10] Cai Y., Wang Y., Shi C., Dai Y., Li F., Xu Y., Zhang P., Kong F., Deng G., Wen Z. (2022). Single-cell immune profiling reveals functional diversity of T cells in tuberculous pleural effusion. J. Exp. Med..

[11] Ura T., Okuda K., Shimada M. (2014). Developments in Viral Vector-Based Vaccines. Vaccines.

[12] Wang S., Liang B., Wang W., Li L., Feng N., Zhao Y., Wang T., Yan F., Yang S., Xia X. (2023). Viral vectored vaccines: Design, development, preventive and therapeutic applications in human diseases. Signal Transduct. Target. Ther..

[13] Zabaleta N., Dai W., Bhatt U., Hérate C., Maisonnasse P., Chichester J.A., Sanmiguel J., Estelien R., Michalson K.T., Diop C. (2021). An AAV-based, room-temperature-stable, single-dose COVID-19 vaccine provides durable immunogenicity and protection in non-human primates. Cell Host Microbe.

[14] Winston S.M., Wiggins K.B., Schultz-Cherry S., Davidoff A.M. (2025). Teaching an old vector new tricks: The surprising versatility of AAV vaccines. J. Virol..

[15] Colon-Cortes Y., Hasan M.A., Aslanidi G. (2020). Intra-tracheal delivery of AAV6 vectors results in sustained transduction in murine lungs without genomic integration. Gene.

[16] Gadenstaetter A.J., Schmutzler L., Grimm D., Landegger L.D. (2022). Intranasal application of adeno-associated viruses: A systematic review. Transl. Res. J. Lab. Clin. Med..

[17] Afkhami S., D’Agostino M.R., Vaseghi-Shanjani M., Lepard M., Yang J.X., Lai R., Choi M.W.Y., Chacon A., Zganiacz A., Franken K. (2023). Intranasal multivalent adenoviral-vectored vaccine protects against replicating and dormant M.tb in conventional and humanized mice. npj Vaccines.

[18] van Doremalen N., Purushotham J.N., Schulz J.E., Holbrook M.G., Bushmaker T., Carmody A., Port J.R., Yinda C.K., Okumura A., Saturday G. (2021). Intranasal ChAdOx1 nCoV-19/AZD1222 vaccination reduces viral shedding after SARS-CoV-2 D614G challenge in preclinical models. Sci. Transl. Med..

[19] Wu S., Huang J., Zhang Z., Wu J., Zhang J., Hu H., Zhu T., Zhang J., Luo L., Fan P. (2021). Safety, tolerability, and immunogenicity of an aerosolised adenovirus type-5 vector-based COVID-19 vaccine (Ad5-nCoV) in adults: Preliminary report of an open-label and randomised phase 1 clinical trial. Lancet Infect. Dis..

[20] Jeyanathan M., Afkhami S., Kang A., Xing Z. (2023). Viral-vectored respiratory mucosal vaccine strategies. Curr. Opin. Immunol..

[21] Elkashif A., Alhashimi M., Sayedahmed E.E., Sambhara S., Mittal S.K. (2021). Adenoviral vector-based platforms for developing effective vaccines to combat respiratory viral infections. Clin. Transl. Immunol..

[22] Jung H.E., Ku K.B., Kang B.H., Park J.H., Kim H.C., Kim K.D., Lee H.K. (2023). Intranasal delivery of an adenovirus-vector vaccine co-expressing a modified spike protein and a genetic adjuvant confers lasting mucosal immunity against SARS-CoV-2. Antivir. Res..

[23] Shepp D.H., Dandliker P.S., Meyers J.D. (1986). Treatment of varicella-zoster virus infection in severely immunocompromised patients. A randomized comparison of acyclovir and vidarabine. N. Engl. J. Med..

[24] Yang H., Lei X., Chai S., Su G., Du L. (2024). From pathogenesis to antigens: The key to shaping the future of TB vaccines. Front. Immunol..

[25] Ernst J.D. (2018). Mechanisms of M. tuberculosis Immune Evasion as Challenges to TB Vaccine Design. Cell Host Microbe.

[26] Chauhan P., Reddy P.V., Singh R., Jaisinghani N., Gandotra S., Tyagi A.K. (2013). Secretory phosphatases deficient mutant of Mycobacterium tuberculosis imparts protection at the primary site of infection in guinea pigs. PLoS ONE.

[27] Puri R.V., Reddy P.V., Tyagi A.K. (2013). Secreted acid phosphatase (SapM) of Mycobacterium tuberculosis is indispensable for arresting phagosomal maturation and growth of the pathogen in guinea pig tissues. PLoS ONE.

[28] Rahlwes K.C., Dias B.R.S., Campos P.C., Alvarez-Arguedas S., Shiloh M.U. (2023). Pathogenicity and virulence of Mycobacterium tuberculosis. Virulence.

[29] Fernandez-Soto P., Bruce A.J.E., Fielding A.J., Cavet J.S., Tabernero L. (2019). Mechanism of catalysis and inhibition of Mycobacterium tuberculosis SapM, implications for the development of novel antivirulence drugs. Sci. Rep..

[30] Zhang W., Dong C., Xiong S. (2024). Mycobacterial SapM hampers host autophagy initiation for intracellular bacillary survival via dephosphorylating Raptor. iScience.

[31] Li J., Feng H., Chen D., Zhang H., Liao Y. (2025). Autophagy in mycobacterial infections: Molecular mechanisms, host-pathogen interactions, and therapeutic opportunities. Front. Cell. Infect. Microbiol..

[32] Ramon-Luing L.A., Palacios Y., Ruiz A., Téllez-Navarrete N.A., Chavez-Galan L. (2023). Virulence Factors of Mycobacterium tuberculosis as Modulators of Cell Death Mechanisms. Pathogens.

[33] Huygen K. (2014). The Immunodominant T-Cell Epitopes of the Mycolyl-Transferases of the Antigen 85 Complex of M. tuberculosis. Front. Immunol..

[34] Tengattini S., Bavaro T., Rinaldi F., Temporini C., Pollegioni L., Terreni M., Piubelli L. (2025). Novel tuberculosis vaccines based on TB10.4 and Ag85B: State-of-art and advocacy for good practices. Vaccine.

[35] Dietrich J., Aagaard C., Leah R., Olsen A.W., Stryhn A., Doherty T.M., Andersen P. (2005). Exchanging ESAT6 with TB10.4 in an Ag85B fusion molecule-based tuberculosis subunit vaccine: Efficient protection and ESAT6-based sensitive monitoring of vaccine efficacy. J. Immunol..

[36] Halbert C.L., Allen J.M., Miller A.D. (2001). Adeno-associated virus type 6 (AAV6) vectors mediate efficient transduction of airway epithelial cells in mouse lungs compared to that of AAV2 vectors. J. Virol..

[37] Limberis M.P., Adam V.S., Wong G., Gren J., Kobasa D., Ross T.M., Kobinger G.P., Tretiakova A., Wilson J.M. (2013). Intranasal antibody gene transfer in mice and ferrets elicits broad protection against pandemic influenza. Sci. Transl. Med..

[38] Limberis M.P., Wilson J.M. (2006). Adeno-associated virus serotype 9 vectors transduce murine alveolar and nasal epithelia and can be readministered. Proc. Natl. Acad. Sci. USA.

[39] Perdomo C., Zedler U., Kühl A.A., Lozza L., Saikali P., Sander L.E., Vogelzang A., Kaufmann S.H., Kupz A. (2016). Mucosal BCG Vaccination Induces Protective Lung-Resident Memory T Cell Populations against Tuberculosis. mBio.

[40] Satti I., Meyer J., Harris S.A., Manjaly Thomas Z.R., Griffiths K., Antrobus R.D., Rowland R., Ramon R.L., Smith M., Sheehan S. (2014). Safety and immunogenicity of a candidate tuberculosis vaccine MVA85A delivered by aerosol in BCG-vaccinated healthy adults: A phase 1, double-blind, randomised controlled trial. Lancet Infect. Dis..

[41] Dietrich J., Andersen C., Rappuoli R., Doherty T.M., Jensen C.G., Andersen P. (2006). Mucosal administration of Ag85B-ESAT-6 protects against infection with Mycobacterium tuberculosis and boosts prior bacillus Calmette-Guerin immunity. J. Immunol..

[42] Bar-Oz M., Meir M., Barkan D. (2022). Virulence-Associated Secretion in Mycobacterium abscessus. Front. Immunol..

[43] Festjens N., Bogaert P., Batni A., Houthuys E., Plets E., Vanderschaeghe D., Laukens B., Asselbergh B., Parthoens E., De Rycke R. (2011). Disruption of the SapM locus in Mycobacterium bovis BCG improves its protective efficacy as a vaccine against M. tuberculosis. EMBO Mol. Med..

[44] Pishesha N., Harmand T.J., Ploegh H.L. (2022). A guide to antigen processing and presentation. Nat. Rev. Immunol..

[45] Hauser B.M., Sangesland M., Denis K.J.S., Windsor I.W., Feldman J., Lam E.C., Kannegieter T., Balazs A.B., Lingwood D., Schmidt A.G. (2021). Rationally designed immunogens enable immune focusing to the SARS-CoV-2 receptor binding motif. bioRxiv.

[46] Osman M.M., Shanahan J.K., Chu F., Takaki K.K., Pinckert M.L., Pagán A.J., Brosch R., Conrad W.H., Ramakrishnan L. (2022). The C terminus of the mycobacterium ESX-1 secretion system substrate ESAT-6 is required for phagosomal membrane damage and virulence. Proc. Natl. Acad. Sci. USA.

[47] Nemes E., Geldenhuys H., Rozot V., Rutkowski K.T., Ratangee F., Bilek N., Mabwe S., Makhethe L., Erasmus M., Toefy A. (2018). Prevention of M. tuberculosis Infection with H4:IC31 Vaccine or BCG Revaccination. N. Engl. J. Med..

[48] Flynn J.L., Chan J., Triebold K.J., Dalton D.K., Stewart T.A., Bloom B.R. (1993). An essential role for interferon gamma in resistance to Mycobacterium tuberculosis infection. J. Exp. Med..

[49] Cooper A.M., Dalton D.K., Stewart T.A., Griffin J.P., Russell D.G., Orme I.M. (1993). Disseminated tuberculosis in interferon gamma gene-disrupted mice. J. Exp. Med..

[50] Wang J., Thorson L., Stokes R.W., Santosuosso M., Huygen K., Zganiacz A., Hitt M., Xing Z. (2004). Single mucosal, but not parenteral, immunization with recombinant adenoviral-based vaccine provides potent protection from pulmonary tuberculosis. J. Immunol..

[51] Santosuosso M., Zhang X., McCormick S., Wang J., Hitt M., Xing Z. (2005). Mechanisms of mucosal and parenteral tuberculosis vaccinations: Adenoviral-based mucosal immunization preferentially elicits sustained accumulation of immune protective CD4 and CD8 T cells within the airway lumen. J. Immunol..

[52] Santosuosso M., McCormick S., Zhang X., Zganiacz A., Xing Z. (2006). Intranasal boosting with an adenovirus-vectored vaccine markedly enhances protection by parenteral Mycobacterium bovis BCG immunization against pulmonary tuberculosis. Infect. Immun..

[53] Stylianou E., Satti I. (2024). Inhaled aerosol viral-vectored vaccines against tuberculosis. Curr. Opin. Virol..

