# Supplementary material for: Intranasal Vaccination with a Recombinant Adeno-Associated Virus Type 6 Encoding SapM Confers Protection Against Tuberculosis
Source: Vaccines (Basel). 2026 Feb 28;14(3):224. doi: 10.3390/vaccines14030224 (PMC13029826; doi:10.3390/vaccines14030224)
Supplement: Supplementary file 1 [file vaccines-14-00224-s001.zip › Figure S1.pdf]

A

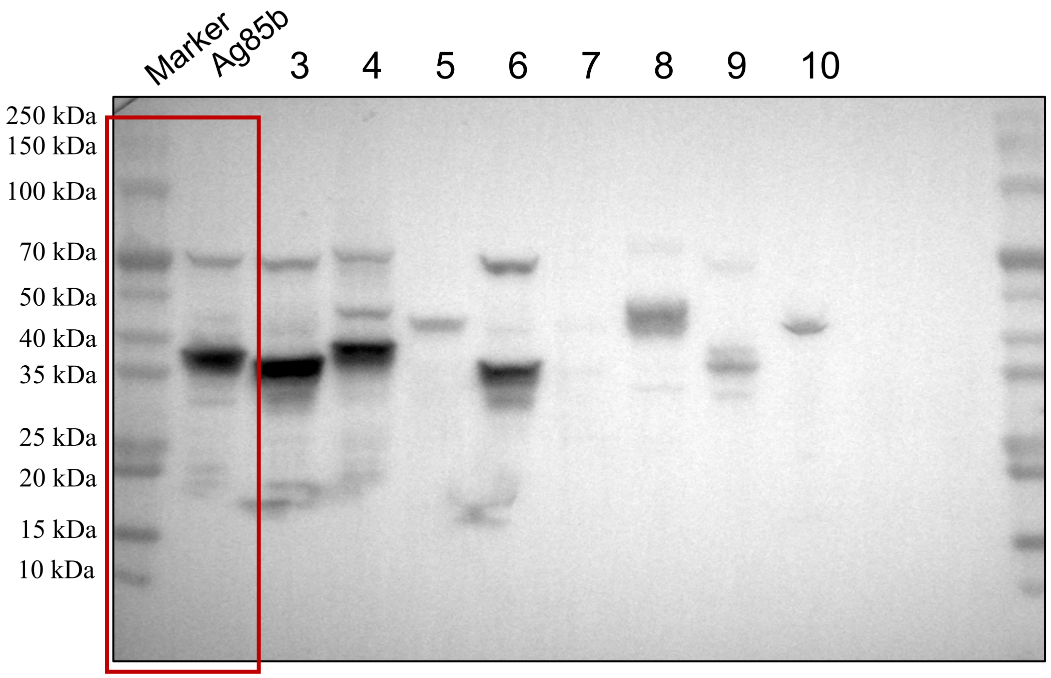

B

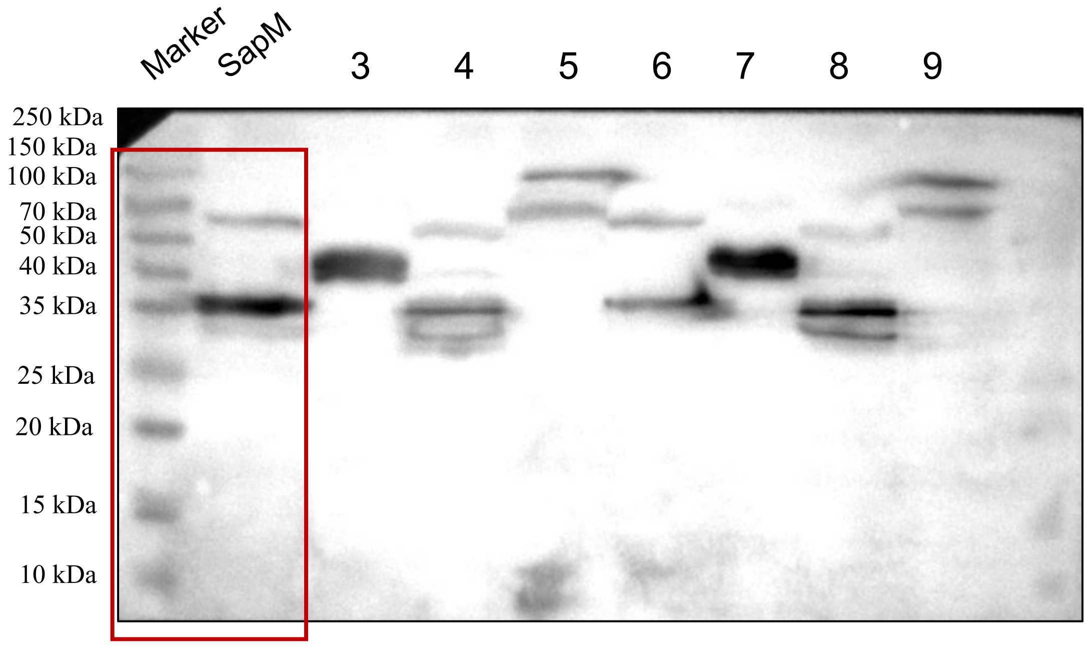

Figure S1. Uncropped original Western blot images for Figure 1G. Representative uncropped Western blots of (A) Ag85b (~37 kDa) and (B) SapM (~35 kDa) expressed in HEK293T cells and probed with anti-3×FLAG. Red boxes indicate the bands presented in Figure 1G. Molecular weight markers (kDa) are indicated.
