# Supplementary material for: Intranasal Vaccination with a Recombinant Adeno-Associated Virus Type 6 Encoding SapM Confers Protection Against Tuberculosis
Source: Vaccines (Basel). 2026 Feb 28;14(3):224. doi: 10.3390/vaccines14030224 (PMC13029826; doi:10.3390/vaccines14030224)
Supplement: Supplementary file 1 [file vaccines-14-00224-s001.zip › Figure S2.pdf]

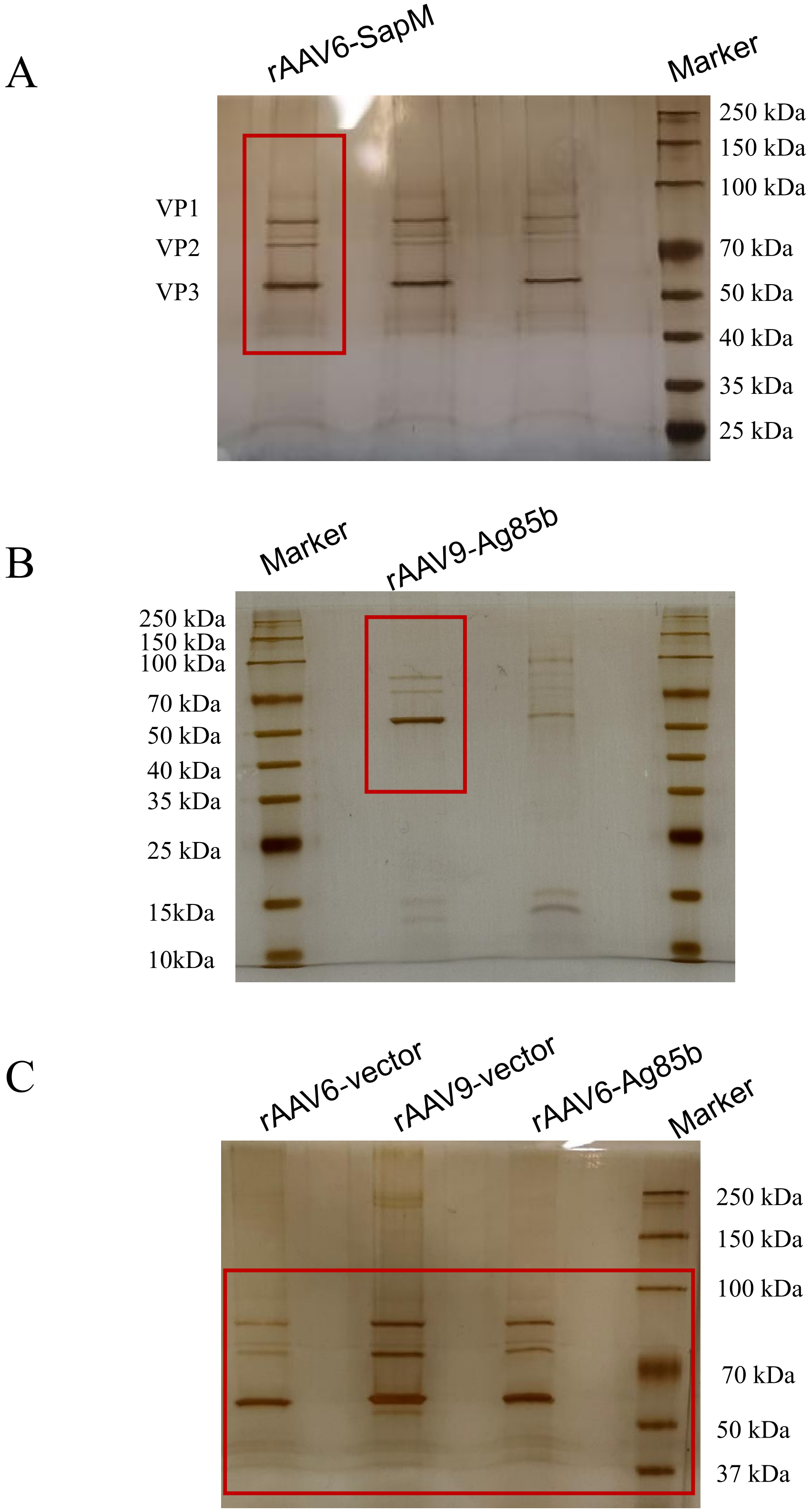

Figure S2. Uncropped original silver-stained gels corresponding to Figure 1H. Representative uncropped silver staining of (A) rAAV6-SapM, (B) rAAV9-Ag85b, and (C) rAAV6-vector, rAAV9-vector and rAAV6-Ag85b. The three major capsid protein bands from top to bottom correspond to VP1 (~87 kDa), VP2 (~72 kDa), and VP3 (~62 kDa). Red boxes indicate the bands presented in Figure 1H. Molecular weight markers (kDa) are indicated.
