# Supplementary material for: Intranasal Vaccination with a Recombinant Adeno-Associated Virus Type 6 Encoding SapM Confers Protection Against Tuberculosis
Source: Vaccines (Basel). 2026 Feb 28;14(3):224. doi: 10.3390/vaccines14030224 (PMC13029826; doi:10.3390/vaccines14030224)
Supplement: Supplementary file 1 [file vaccines-14-00224-s001.zip › Figure S3.pdf]

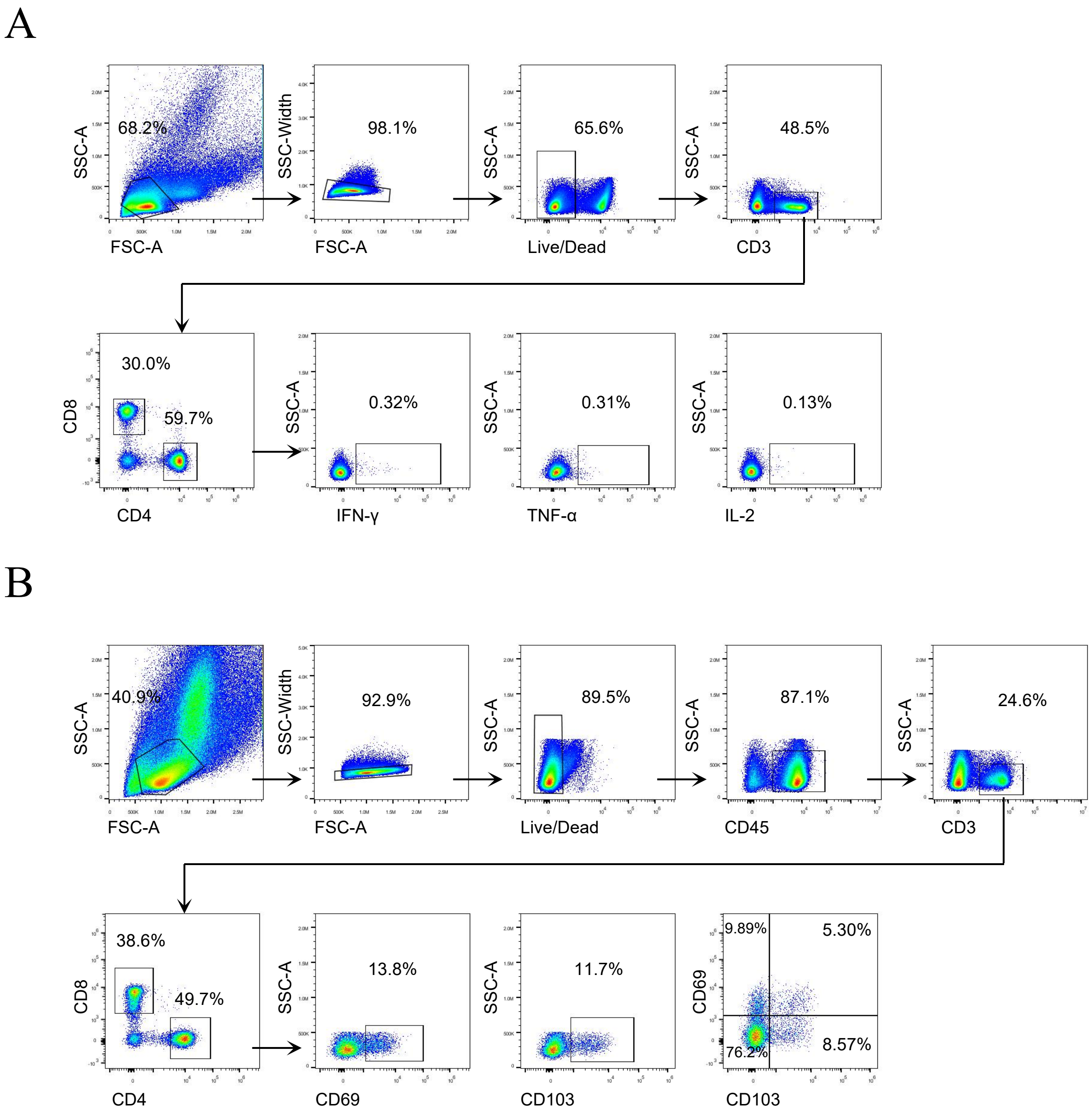

Figure S3. Flow cytometry gating strategies for splenocytes and lung cells. (A) Gating for cytokine production in splenocytes. At 6 weeks post-immunization, splenocytes were stimulated ex vivo with H37Rv lysate. Lymphocytes were identified by FSC-A/SSC-A, followed by singlet discrimination and dead cell exclusion. Within the live CD3⁺ T cell population, CD4⁺ and CD8⁺ subsets were identified. The frequencies of IFN-γ, TNF-α, and IL-2 producing cells were analyzed within the gated CD4⁺ T cell population. (B) Gating for lung tissue-resident memory T cells. At 6 weeks post-immunization, lung mononuclear cells were analyzed without further stimulation. Following sequential gating of lymphocytes, singlets, and live cells, CD3⁺ T cells were identified within the CD45⁺ leukocyte population. T cells were further subdivided into CD4⁺ and CD8⁺ populations. The expression of resident markers CD69 and CD103 was analyzed within both CD4⁺ and CD8⁺ T cell subsets (representative plots for CD4⁺ T cells are shown, CD8⁺ gating was performed similarly). Numbers in the plots represent the percentage of cells within the indicated gates.
