# Supplementary material for: Intranasal Vaccination with a Recombinant Adeno-Associated Virus Type 6 Encoding SapM Confers Protection Against Tuberculosis
Source: Vaccines (Basel). 2026 Feb 28;14(3):224. doi: 10.3390/vaccines14030224 (PMC13029826; doi:10.3390/vaccines14030224)
Supplement: Supplementary file 1 [file vaccines-14-00224-s001.zip › Figure S4.pdf]

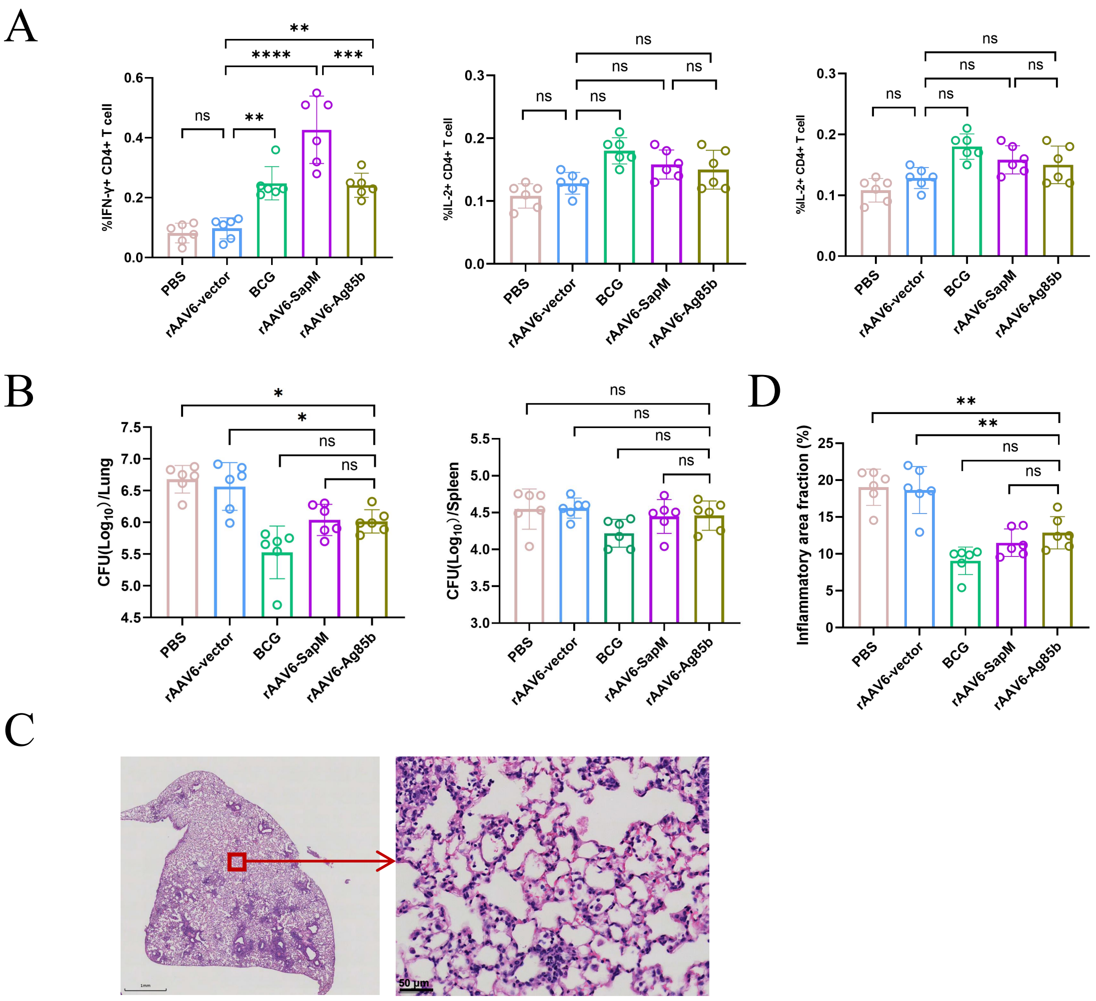

Figure S4. Immunogenicity and protective efficacy of rAAV6-Ag85b. (A) At 6 weeks post-immunization, splenocytes were stimulated ex vivo with H37Rv lysate, the frequency of IFN-γ, TNF-α and IL-2 $CD4^+$ T cells was quantified by ICS. (B) Bacterial burdens in the lungs and spleen at 4 weeks post-challenge. (C) Representative lung H&E sections 4 weeks post-H37Rv challenge. Scale bars equal 1mm. Outlined areas in the main images indicate enlarged regions, with a scale bar of 50 µm. (D) Quantification of lung inflammation was performed on whole-slide images, calculated as the percentage of the inflamed area relative to the total lung tissue area using Qupath software (V0.5.1). Data were plotted as mean ± SEM (n = 6). Group differences were analyzed using one-way ANOVA with Tukey, ns, not significant; $*p< 0.05$, $**p< 0.01$; $***p < 0.001$; $****p < 0.0001$
