# Supplementary material for: Intranasal Vaccination with a Recombinant Adeno-Associated Virus Type 6 Encoding SapM Confers Protection Against Tuberculosis
Source: Vaccines (Basel). 2026 Feb 28;14(3):224. doi: 10.3390/vaccines14030224 (PMC13029826; doi:10.3390/vaccines14030224)
Supplement: Supplementary file 1 [file vaccines-14-00224-s001.zip › Figure S5.pdf]

A

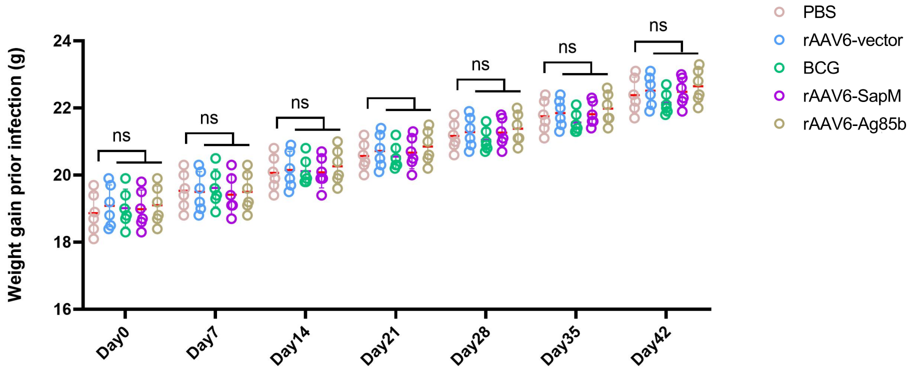


B

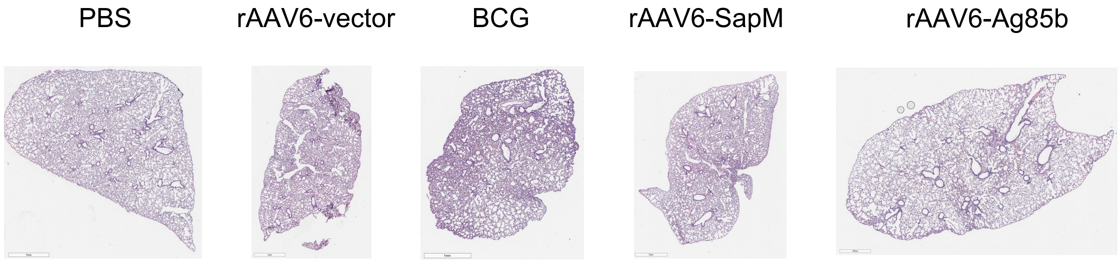


**Figure S5. Safety assessment of rAAV6 immunization prior to *Mtb* challenge.** (A) C57BL/6 mice were weighted weekly (Day 0-42) to monitor their weight gain before *Mtb* infection (n=6). no significant differences in body weight were observed among groups (ns). (B) Representative HE stained lung sections collected 6 weeks after immunization (pre-challenge) from the indicated groups. No apparent increase in lung inflammation or tissue pathology was observed in rAAV6-immunized mice compared with PBS or vector controls.Group differences were analyzed using Two-way repeated-measures ANOVA (group × time), ns, not significant.
